# MALDI-TOF MS Indirect Beta-Lactamase Detection in Ampicillin-Resistant *Haemophilus influenzae*

**DOI:** 10.3390/microorganisms11041018

**Published:** 2023-04-13

**Authors:** Lukas Hleba, Miroslava Hlebova, Eva Kovacikova, Anton Kovacik

**Affiliations:** 1 Institute of Biotechnology, Faculty of Biotechnology and Food Sciences, Slovak University of Agriculture in Nitra, Tr. Andreja Hlinku 2, 949 76 Nitra, Slovakia; 2 Department of Biology, Faculty of Natural Sciences, University of Ss. Cyril and Methodius, Nám. J. Herdu 2, 917 01 Trnava, Slovakia; 3 AgroBioTech Research Centre, Slovak University of Agriculture in Nitra, Tr. A. Hlinku 2, 949 76 Nitra, Slovakia; 4 Institute of Applied Biology, Faculty of Biotechnology and Food Sciences, Slovak University of Agriculture in Nitra, Tr. A. Hlinku 2, 949 76 Nitra, Slovakia

**Keywords:** mass spectrometry, *Haemophilus influenzae*, resistance, MIC, β-lactamase

## Abstract

Rapid identification of beta-lactamase-producing strains of *Haemophilus influenzae* plays key role in diagnostics in clinical microbiology. Therefore, the aim of this study was the rapid determination of beta-lactamase’s presence in *H. influenzae* isolates via indirect detection of degradation ampicillin products using MALDI-TOF MS. *H. influenzae* isolates were subjected to antibiotic resistance testing using disk diffusion and MIC methodologies. Beta-lactamase activity was tested using MALDI-TOF MS, and results were compared to spectral analysis of alkaline hydrolysis. Resistant and susceptible strains of *H. influenzae* were distinguished, and strains with a high MIC level were identified as beta-lactamase-producing. Results indicate that MALDI-TOF mass spectrometry is also suitable for the rapid identification of beta-lactamase-producing *H. influenzae*. This observation and confirmation can accelerate identification of beta-lactamase strains of *H. influenzae* in clinical microbiology, which can have an impact on health in general.

## 1. Introduction

*Haemophilus influenzae* (*H. influenzae*) is a Gram-negative microorganism that commonly colonizes the upper respiratory tract in humans, especially children. It can cause various invasive diseases, such as pneumonia, sepsis and meningitis [1,2]. Prompt antibiotic treatment is key in the management of infection caused by *H. influenzae*. β-lactam antibiotics such as ampicillin are commonly used to treat infections caused by *H. influenzae*, but resistant isolates often occur [3]. For example, the number of isolates resistant to ampicillin increased from 8.6% in 2010 to 22.1% in 2019 in the Czech Republic [4], from 14.9% (2007–2009) to 24% (2013) in Italy [5,6,7], to 57.6% in Asia, 70.4–73.3% in North America and the Middle East, 80.7–86.1% in Europe, Latin America and Africa and from 13.5% (2007–2010) to 19% (2011–2014) in Canada [8]. Due to the widespread use of antibiotics, antimicrobial resistance, particularly resistance to ampicillin, continues to increase, and added resistance to macrolides, fluoroquinolones and other beta-lactams, including carbapenems, is increasing [9]. Reduced susceptibility to ampicillin may result from a variety of mechanisms. Isolates resistant to β-lactams can produce β-lactamase or have substitutions in the penicillin-binding protein (PBP3, encoded by the *fts*I gene), leading to resistance to penicillin and eventually to cephalosporins in resistant β-lactamase-negative isolates. In addition, isolates with both mechanisms (β-lactamase production and altered PBP3 with a reduced affinity for β-lactams) are now appearing that are also resistant to other antibiotics, such as amoxicillin/clavulanate [1,2]. Antibiotics have extended human life by preventing people from getting infectious diseases. However, drug use has led to a drastic increase in antimicrobial resistance (AMR) [10]; therefore, a rapid and accurate diagnosis of antimicrobial susceptibility is needed in order to optimize the therapeutic strategy for patients with infectious diseases and to control infection in healthcare facilities. When it comes to AMR of the bacteria causing the infection, doctors need at least three pieces of information from clinical microbiology laboratories: 1. whether the patient is infected with bacteria, 2. if so, which bacterial species they are infected with and 3. which antibiotic is the right one to use for treating the patient. Fast and correct diagnostics helps doctors to choose an appropriate treatment regimen, improve patient outcomes, shorten patient hospitalizations and reduce the possibility of pathogen spread [11]. The gold standards of antimicrobial susceptibility testing (AST) are based on bacterial growth methods that determine the diameter of the inhibition zone around the antimicrobial discs on the surface of the agar or the minimum inhibitory concentration of the antimicrobial drug according to the guidelines standardized by Clinical Laboratory Standards in the USA or EUCAST in Europe [12,13]. The main drawback of these methods is that their implementation takes several days, which prevents the rapid development of correct treatment strategies for the patient. Identification is mostly based on molecular–biological methodologies focused on determining the genotype. However, they also have many limitations, such as being time-consuming, the fact that the genotype does not always correspond to the phenotype and the need for experienced experts to interpret the data. Finally, this technique is laborious and cost-ineffective, which prevents its widespread use in clinical microbiology laboratories [14]. Among the various techniques that have been proposed for the rapid diagnosis of AMR and identification of microorganisms, the matrix-assisted laser desorption–time-of-flight mass spectrometry (MALDI-TOF MS) method is already widely used in the clinical setting for rapid species identification of organisms [15,16]. Antimicrobial resistance to several antibiotics belonging to different classes has been successfully tested for using MALDI-TOF MS in a variety of clinically relevant bacterial species, including members of the *Enterobacteriaceae* family, non-fermenting Gram-negative bacteria, Gram-positive cocci, anaerobic bacteria and mycobacteria, thus opening the field to further clinically important developments [17]. Early detection of drug resistance using MALDI-TOF MS may be particularly useful for clinicians in streamlining antibiotic treatment to achieve a better outcome in patients with systemic infection in all cases where rapid and effective antibiotic treatment is essential to preserve organ function and/or patient survival. The application of MALDI-TOF MS technology supplies a new, exact, and robust tool enabling rapid and reliable microbial and AMR identification [16,18].

Therefore, the aim of this study was the rapid determination of beta-lactamase’s presence in *H. influenzae* isolates through indirect detection of degradation ampicillin products using MALDI-TOF MS.

## 2. Materials and Methods

### 2.1. Isolation and Identification of Haemophilus influenzae

Swabs from the upper respiratory tract (30 samples) were the source of isolates. Samples were collected with sterile cotton swabs and transferred to Stuart’s transport medium (Remel, Kansas City, AR, USA) to the Department of Microbiology, FBFS, in SUA in Nitra. Swabs were cut and inserted into sterile physiological solution. Samples were vortexed, and 100 µL of bacterial solution was transferred to Columbia agar plates with 5% sheep blood containing diluted ampicillin (Sigma Aldrich, München, Germany) at a concentration of 1 mg/L according to ECOFF (EUCAST). Cultivation was performed at 35 °C in aerobic modified conditions with 5% CO_2_ overnight. Simultaneously, another colony was cultivated in the same conditions but without the presence of antibiotics in the nutrient medium to obtain sensitive strains. Purification of bacterial colonies was achieved by picking out potential *Heamophilus* colonies growing inside the hemolytic zones of streptococci and transferring them to the chocolate agar containing the X factor (hemin) (Sigma Aldrich, München, Germany). Identification of bacterial isolates was performed using MALDI-TOF MS (Bruker Daltonik GmbH, Bremen, Germany) with the associated software Biotyper (Bruker Daltonik GmbH, Bremen, Germany). A detailed description of the protein isolation and identification procedure is provided in a previous study [19].

### 2.2. Susceptibility Testing of H. Influenzae

Antibiotic susceptibility testing was performed on chocolate agar including X factor (Sigma Aldrich, München, Germany). Ampicillin paper disks filled with 2 µg of ampicillin (Oxoid, Hampshire, UK) were applied to agar plates. Bacterial strains were incubated at 35 °C under a humidified atmosphere containing 5% CO_2_ for 16–20 h. All resistance strains were subjected to MIC determination using an E-test (antibiotic strips M.I.C. Evaluator) (Oxoid, Hampshire, UK) with a concentration ranging from 0.016 to 256 µg/mL. For MIC determination, the same conditions were used. After incubation, the inhibition zones around the disks and the strips were assessed according to EUCAST [20]. Susceptible strains were transferred to chocolate agar containing X factor and recultivated until use.

### 2.3. MALDI TOF Ampicillin Analysis

Pure ampicillin, in the form of sodium salt (Sigma Aldrich, München, Germany), was used in this experiment. For MALDI TOF MS detection, 20 mM Tris-HCl buffer (pH 7) containing 20 µg/mL of ampicillin sodium salt was applied [21,22]. α-cyano-4-hydroxycinnamic acid (CHCA) was used as a matrix for MALDI TOF MS. The matrix was diluted in organic solvent containing 500 µL of 100% acetonitrile, 475 µL of distilled water and 25 µL of 100% tri-fluoracetic acid (all chemicals were of MS purity and obtained from Sigma Aldrich, Germany). One microliter of ampicillin diluted in Tris-HCl (pH 7) was transferred to the target plate (Bruker Daltonics, Bremen, Germany, MSP Target plate) and dried at room temperature. Dried samples were covered by a CHCA matrix. Mass spectra were collected using a Microflex LT mass spectrometer (Bruker Daltonics, Germany) controlled with the flexControl 3.0 software (Bruker Daltonics, Germany). MALDI TOF MS operated in positive linear ion mode and spectra were collected randomly by 500 laser shots and in a range from 300 to 500 *m*/*z*. The settings of device parameters were described previously in study by Hleba et al. [21]. Alkaline hydrolysis using 100 mM NaOH of ampicillin served as a comparative model for obtaining mass spectra of ampicillin degradation products [23]. Additionally, alkaline hydrolysis, as previously mentioned, was used for the calibration of MALDI TOF MS with a matrix for this experiment.

### 2.4. Ampicillin Hydrolysis Assay

Resistant and susceptible *H. influenzae* bacterial strains isolated from the upper respiratory tract were incubated overnight in chocolate agar containing X factor (Sigma Aldrich, München, Germany) in the same conditions to increase the number of viable colonies. After incubation, colonies were transferred into physiological solution containing 20 mM Tris-HCl (pH 7) and 150 mM NaCl to acquire solution with 4 McF°. Bacterial solution was mixed, and the pellet was separated from the supernatant via centrifugation (3 min at maximum speed). A total of 100 µL 20 mM Tris-HCl buffer (pH 7) with 20 µg/mL ampicillin was added to the pellet and mixed via pipetting. The resulting suspension was incubated at 35 °C for 3 h. After incubation, the suspension was centrifuged and 1 µL of supernatant was analyzed using MALDI TOF MS. All samples were tested in triplicate.

### 2.5. Spectral Analysis

Spectral analysis was performed using flexAnalysis 3.0 software (Bruker Daltonics, Bremen, Germany). Obtained peaks were processed using the centroid detection algorithm with a signal-to-noise threshold of 1, a relative intensity threshold of 0%, a minimum intensity threshold of 0, a peak width of 0.2 *m*/*z* and a cycle of 1. Theoretical peaks of ampicillin, ampicillin sodium salt, degradation products and its sodium salts were compared with our detected masses with ±0.6 *m*/*z*.

## 3. Results

### 3.1. Antimicrobial Susceptibility Testing

Rapid detection of ampicillin-resistant *H. influenzae* isolates was the focus of this study, as well as indirect detection of ampicillin degradation products using MALDI-TOF MS. In the first part of this study, AMP resistance was monitored in all (30) obtained isolates of *H. influenzae*. All isolated resistant strains of *H. influenzae* showed an inhibition zone smaller than 18 mm. Additionally, our MALDI-TOF mass spectra confirmed production of beta-lactamase via the indirect method of degradation product detection.

The distribution of MIC is shown in Table 1. Two resistant isolates were detected, which showed different resistance characteristics. On the other hand, 28 susceptible isolates were used as a comparative model.

### 3.2. MALDI-TOF MS Ampicillin Analysis and Alkaline Hydrolysis

**Figure 1:**
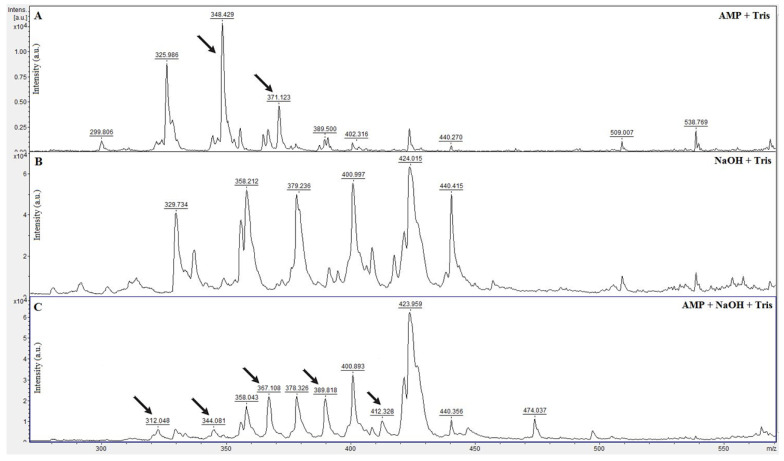
Mass spectra of ampicillin in Tris-HCl (**A**) with spectra of ampicillin (348 *m*/*z*), its sodium salt (371 *m*/*z*), Tris-HCl and NaOH (**B**) and alkaline hydrolysis of ampicillin with NaOH in Tris-HCl (**C**) with spectra of spontaneous decarboxylated ampicillin (323 *m*/*z*), decarboxylated ampicillin sodium salt (344 *m*/*z*), ampicillin with a disrupted amide bond (367 *m*/*z*), its sodium salt (389 *m*/*z*) and its disodium salt (412 *m*/*z*). Important peaks are marked by arrows.

In this experiment, CHCA (α-cyano-4-hydroxycinnamic acid) with spectra at 189 *m*/*z* (monomer) and 378 *m*/*z* (dimer) were identified and used as a spectral calibrant. The selection of a suitable matrix was performed based on previous successful studies [21,24], wherein the authors recommended several matrices. Furthermore, for the visualization of ampicillin and its degradation products, it is better to use CHCA due to the reduction in spectral noise. Additionally, the concentration of ampicillin used, which could be dissolved in Tris-HCl (pH 7) and still be detected via MALDI-TOF MS, was based on previous study. A comparative spectral model of alkaline ampicillin hydrolysis was proposed, where degradation products are the same as in ampicillin hydrolysis by beta-lactamase. The results of alkaline hydrolysis are shown in Figure 1.

Obtained mass spectra of pure ampicillin showed two peaks with relative molecular weights of 348 *m*/*z* for a molecule of ampicillin and 371 *m*/*z* for its sodium salt dissolved in Tris-HCl (pH7). On the other hand, alkaline hydrolysis of ampicillin showed different peaks that represented its degradation products. Alkaline sodium hydroxide, such as the beta-lactamase enzyme, cleaves the ampicillin beta-lactam core to form ampicillin with a disrupted amide bond with a relative molecular weight of 367 *m*/*z*. Therefore, three peaks were observed in the mass spectra, representing ampicillin with a disrupted amide bond (367 *m*/*z*), its sodium salt (389 *m*/*z*) and disodium salt (412 *m*/*z*). In addition, after the hydrolysis of ampicillin and the disrupted beta-lactam core of the molecule, spontaneous decarboxylation of hydrolytically cleaved ampicillin occurred to form decarboxylated ampicillin and its sodium salt with relative molecular weights of 323 *m*/*z* and 344 *m*/*z*.

### 3.3. Ampicillin Hydrolysis by H. Influenzae

**Figure 2:**
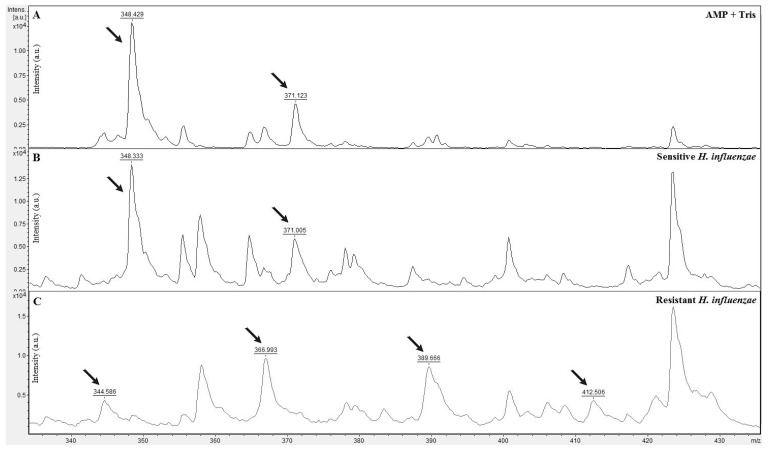
Mass spectra of ampicillin hydrolysis by beta-lactamase of *H. influenzae*. (**A**) Ampicillin (348 *m*/*z*) and its sodium salt (371 *m*/*z*), (**B**) non-beta-lactamase-producing *H. influenzae* and (**C**) beta-lactamase-producing *H. influenzae* with degradation products in spectra: decarboxylated ampicillin sodium salt (344 *m*/*z*), ampicillin with a disrupted amide bond (366 *m*/*z*), its sodium salt (389 *m*/*z*) and its disodium salt (412 *m*/*z*). Important peaks are indicated by arrows.

In this study, the hydrolysis of ampicillin by *H. influenzae* was detected in two *H. influenzae* strains, which showed resistance against ampicillin in previously developed study methods (disk diffusion and MIC methodology). Other isolated strains were used as a comparative spectral model for comparison between resistant (able to hydrolyze ampicillin) and susceptible (not able to hydrolyze ampicillin) strains. Spectral analysis of beta-lactamase hydrolysis using MALDI-TOF MS after 3 h of incubation of resistant *H. influenzae* with ampicillin in Tris-HCl (pH 7) showed the following mass spectra: 344 *m*/*z* represents decarboxylated ampicillin sodium salt, ampicillin with a disrupted amide bond (366 *m*/*z*), its sodium salt (389 *m*/*z*) and its disodium salt (412 *m*/*z*). Decarboxylated pure ampicillin was not detected in this case. As seen in Figure 2, sensitive *H. influenzae* could not cleave the beta-lactam core of the ampicillin molecule and the spectrum remained the same as that of the original antibiotic with two peaks, which represented ampicillin (348 *m*/*z*) and its sodium salt (371 *m*/*z*). Results presented in Figure 2 are the average of multiple repetitions of the spectra obtained from resistant and susceptible *H. influenzae*.

## 4. Discussion

Currently, AMR (antibiotic multidrug resistance) is considered a major public health problem and threatens human health and patient treatment [14]. In clinical practice, many diseases are caused by pathogens that are also resistant to antibiotics. This level of resistance increases over time, which can pose a significant threat to infection control in patients, especially in surgery or intensive care units [17]. One important class of antimicrobial-resistant bacteria is β-lactamase-producing bacteria, which can hydrolyze β-lactam antibiotics such as *H. influenzae* resistant to ampicillin [25]. When treating diseases caused by *H. influenzae*, doctors need fast and reliable methods to identify the pathogens, as well as rapid detection of antibiotic resistance, so that appropriate and timely treatment can be administered. Therefore, MALDI-TOF MS provides an excellent tool for quick, time- and cost-effective identification of pathogenic organisms and also enables the detection of antibiotic resistance, including the identification of resistance mechanisms in various bacteria [11,26,27,28]. This study was therefore aimed at testing ampicillin-resistant strains of *H. influenzae*. Firstly, it was necessary to select resistant and sensitive strains. The strains were tested for their sensitivity to ampicillin using an EUCAST-established ECOFF threshold at the level of 1 mg/L for ampicillin [29]. Therefore, 1 mg/L of ampicillin was added into nutrient media for resistant strain selection. After adding ampicillin into the agar concentrated based on the ECOFF information provided in EUCAST (ECOFF for *H. influenzae*) in the initial cultivation phase, only resistant bacterial colonies grew. However, it should be mentioned that it was necessary to isolate all strains of *H. influenzae* (susceptible and resistant) for the purposes of this experiment, because the aim of the experiment was to investigate the hydrolytic cleave reaction using MALDI-TOF MS in ampicillin-resistant *H. influenzae*, and susceptible strains served as a comparative model. A comparative model based on the comparison of degradation products of antibiotics after alkaline hydrolysis and hydrolysis with beta-lactamase using MALDI-OF MS has already been used by several authors [21,24,25,30,31,32]. The susceptibility of tested *H. influenzae* strains to antibiotics was performed on chocolate agar including X factor in this study. Nørskov-Lauritsen et al. [33] used the same agar for susceptibility testing of *H. influenzae* and recommended chocolate agar containing X factor (hemin), because all tested strains reached maturity. Resistant *H. influenzae* grew with double zones around the ampicillin disk, and previous studies confirmed double zones around ampicillin with connection to beta-lactamase production. Other studies also found that the disk diffusion methods were able to differentiate between low-BLNAR *H. influenzae* from the wild-type population with sensitivities ranging from 87% to 98% and specificities from 96% to 99%. They recommended using cefaclor as a superior antibiotic to ampicillin. The opposite case was made up of susceptible strains. Additionally, minimal inhibition concentration testing using an M.I.C.E strip in resistant strains showed an MIC of more than 1 mg/L, as well as one sample with 8 mg/L and one with 16 mg/L. Our results agree with the authors Kayser et al. [34], who also determined the susceptibility of ampicillin-resistant isolates of *H. influenzae* to ampicillin. They recorded the same results; for one isolate, an MIC of 16 mg/L was found, and for four other isolates, the MIC ranged from 8 to 4 mg/L, similarly to our study. Additionally, in a study by Bell and Plowman [35], the authors found that in four of the β-lactamase-positive strains, the MIC of ampicillin exceeded 8 mg/l. However, in their study, ampicillin was tested in the concentration range of 8 to 0.25 mg/L. For example, the authors Bajanca-Lavdo et al. [36] tested 96 isolates of β-lactamase-producing strains of *H. influenzae* resistant to ampicillin and reported a lower MIC (4 mg/L) for tested strains. On the contrary, the authors Thiên-Trí Lâm et al. [37] recorded a much higher MIC for tested isolates of *H. influenzae*. They found that among all tested clinical isolates of *H. influenzae* (82), up to 65 isolates showed β-lactamase production. Of these, one isolate showed an MIC of 24 mg/L, one an MIC of 32 mg/L and four isolates showed an MIC of ≥256 mg/L. In our study, the tested strains of *H. influenzae* showed zones that were smaller than 18 mm. The boundary between resistance and susceptibility was established by EUCAST [20] at the level of 18 mm using the disk diffusion method. Tristam [38] described beta-lactamase-positive ampicillin-resistant (BLPAR) strains of *H. influenzae* as generally have relative high ampicillin MICs, but isolates are sensitive to cephalosporins and co-amoxiclav. On the contrary, beta-lactamase-negative ampicillin-resistant (BLNAR) strains have relatively low ampicillin MICs and low levels of resistance or decreased sensitivity to cephalosporins and co-amoxiclav due to altered PBP_3_. In this study, it was not essential to distinguish BLPAR from BLNAR strains of *H. influenzae*, since beta-lactamase activity was detected using indirect detection of ampicillin degradation products, which proves the presence of beta-lactamase. Indirect methodology for beta-lactamase detection based on observing degradation products of antibiotics from penicillins, cephalosporins, carbapenems and monobactams has been described in several species of bacteria, such as *E. coli* [21,39], *Klebsiella pneumoniae* [24,40,41], *Pseudomonas* spp. [42,43], *Prevotella* species [44] and other clinically important bacterial species [41]. However, no reference of MALDI-TOF detection of beta-lactamase hydrolysis of any antibiotic including ampicillin was found in the available literature. Therefore, *H. influenzae* became the main subject of this study.

In next step, the ampicillin analysis and alkaline hydrolysis were carried out using MALDI-TOF MS. Many studies [45,46,47] described the mechanisms of beta-lactam antibiotics hydrolysis in acidic and alkaline environments in connection with the human body and antibiotic action. Hydrolysis can be a degradation pathway for some organic compounds, especially esters and amides. The products of this hydrolysis may be less bioaccumulative than the parent compound because they are more polar, making them more soluble in water [48,49]. Common antibiotic hydrolysis reaction sites include labile carbonyl groups such as lactones and lactams or esters [50]. Mitchells et al. [49] investigated the effect of pH and temperature on the hydrolysis of three beta-lactam ATBs: ampicillin, cephalothin and cefoxitin. They found that the degree of antibiotic hydrolysis was highly dependent on pH, temperature and the presence of hydrolytically sensitive functional groups in the antibiotic structure. Hydrolysis of the antibiotic was independent of pH from pH 4 to 8. Half-lives at pH 7 and 25 °C for cephalothin, cefoxitin and ampicillin were 5.3, 9.3 and 27 days, respectively. The hydrolysis half-life of ampicillin was longer than that of cephalothin and cefoxitin under acidic and ambient conditions. They also found that deionized water buffer had a minimal effect on hydrolysis. All authors agree that alkaline hydrolysis and its degradation products are consistent with the beta-lactamase hydrolysis process of microorganisms [21,23,44,51,52,53]. Therefore, alkaline hydrolysis was used as a comparative spectral model. Three peaks representing ampicillin with a broken amide bond (367 *m*/*z*), its sodium salt (389 *m*/*z*) and its disodium salt (412 *m*/*z*) were observed in the obtained mass spectra. After hydrolysis of ampicillin and breaking of the beta-lactamase core, decarboxylated ampicillin and its sodium salt were also observed. The intensity and height of the peaks are probably associated with the amount of degradation products, the antibiotics themselves and their sodium salts at the time of obtaining the spectra. As in the previous statement, all authors describe the same or very similar alkaline hydrolysis reaction products in their studies [21,23,43,51,52,53]. In our previous study [51], similar results were obtained for the alkaline hydrolysis of ampicillin (ampicillin with an interrupted amide bond (366 *m*/*z*) by alkaline hydrolysis (NaOH), its sodium salts (monosodium salt 389 *m*/*z*, disodium salt 412 *m*/*z*), spontaneous decarboxylated ampicillin (323 *m*/*z*) and decarboxylated ampicillin sodium (344 *m*/*z*). Not all authors were able to detect all the ampicillin degradation products listed above. For example, Toprak et al. [44] used alkaline hydrolysis as a comparative model for beta-lactamase-positive H. influenzae and B. fragilis and were able to detect the presence or absence of ampicillin only in the spectra. In their study, the reason the authors used the same 100 mM NaOH solution with 10 mM ampicillin and the same matrix (CHCA = α-cyano-4-hydroxycinnamic acid) was not given. Differences were found when different MAL-DI-TOF MS instruments were used. Whether these were due to a detection error or the wrong methodological procedure was not expanded upon. However, it was confirmed that the main ampicillin peak disappeared from the spectrum after alkaline hydrolysis. It is also important to emphasize that not all other researchers need to find the exact number of peaks in the mass spectrum or the same amount of degradation products. Much depends on the chemicals used, the reaction time and other internal or external conditions. On the other hand, Sparbier et al. [31] reported the calculated masses of ampicillin degradation products as hydrolyzed ampicillin (368.4 *m*/*z*), hydrolyzed ampicillin sodium (390 *m*/*z*), hydrolyzed ampicillin disodium (412 *m*/*z*) and spontaneously decarboxylated ampicillin (324 *m*/*z*). However, they did not describe the product of alkaline hydrolysis of ampicillin (decarboxylated sodium salt of ampicillin) that was detected with a molecular weight of 344 *m*/*z* during the practical implementation of the experiment in this study. Thus, small deviations in the mass spectrum occur in all studies, which are related to the measurement and setting of the MALDI-TOF instrument itself, its condition, laser intensity and other parameters. However, all of them mention this fact in the methodology as a possible cause of deviation in the shift of the spectrum.

Resistance to ampicillin in *Haemophilus influenzae* is based mainly on two types of mechanisms. One of the mechanisms of resistance is the production of beta-lactamases, specifically TEM-1 [54] and ROB-1 [55] beta-lactamase. The second type of resistance is caused by an alteration in the PBP3 protein. However, in both cases, there is a decrease in affinity to ampicillin and other beta-lactam antibiotics [56,57,58]. Strains of *H. influenzae* with beta-lactamase production are generally called BLPAR (beta-lactamase-producing, ampicillin-resistant). Strains with altered PBP3 (penicillin-binding protein) are called BLNAR (beta-lactamase-nonproducing, ampicillin-resistant). In principle, during the hydrolytic cleavage of ampicillin, degradation products are formed, which can be detected in the mass spectrum using MALDI-TOF MS. Hydrolysis of ampicillin by MALDI TOF MS can thus be detected only in the case of BLPAR strains, because these *H. influenzae* strains produce beta-lactamases [59].

Spectral analysis of beta-lactamase hydrolysis using MALDI-TOF MS showed four mass spectra, namely decarboxylated ampicillin sodium (344 *m*/*z*), ampicillin with disrupted amide bond (366 *m*/*z*), its sodium salt (389 *m*/*z*) and its disodium salt (412 *m*/*z*). However, decarboxylated pure ampicillin was not detected. In principle, all authors [21,23,28,29,31,39,43,54] describe the same hydrolysis products using microbial beta-ta-lactamase. The difference in degradation products is apparent only when using other antibiotics or pure ampicillin without sodium salt. Despite the fact that MALDI-TOF MS is very often used in larger clinical laboratories precisely for the rapid and accurate identification of microorganisms, it also has some limitations [17]. The main limitation in the identification of pathogens is the coverage of the spectral database because the coverage of microbial species in the reference database determines the range of applications for pathogen identification using MALDI-TOF MS [60]. In addition to the coverage of databases, there are also other limitations, such as sample preparation for MALDI-TOF analysis, as purified individual colonies obtained via bacterial culture are needed to achieve high accuracy of MALDI-TOF MS analysis. As possible solutions to this limitation, various new sample pretreatment procedures could serve to reduce the time needed or the culture itself, new matrices or methods could be developed to improve the surface to obtain additional molecular information, new algorithms could be established for data analysis, which would enable the direct identification of bacteria from mixed cultures, etc. [17,27,61]. Additionally, the identification of AMR is not always completely accurate and therefore it is necessary to carry out an ATB sensitivity test (AST) [11,17,25]. Therefore, there is room for improving the detection of enzymes that degrade antibiotics, for example, by creating a database of protein fingerprints of antibiotic-resistant isolates. [11]. For this reason, it is very important that scientists continue to devote themselves to research and experiments carried out on MALDI TOF MS and that the limitations associated with MALDI TOF MS are reduced or eliminated, thus enabling the expansion of its use in clinics not only for the identification of pathogens but also for the analysis of AMR pathogens.

## 5. Conclusions

Based on the results of MALDI-TOF analysis, it can be concluded that it is probably possible to distinguish between beta-lactamase-producing and non-producing strains of *H. influenzae* on the basis of the degradation products of ampicillin. The preparation of the experiment is very similar to the steps carried out in a previous study on microorganisms with specific modifications regarding *H. influenzae* cultivation. Additionally, this observation and confirmation can accelerate the identification of beta-lactamase strains of *H. influenzae* in clinical microbiology, which can have an impact on human health in general. However, larger studies are needed to completely confirm our conclusions.

## Figures and Tables

**Table 1:** MIC distribution and antimicrobial susceptibility of *H. influenzae* isolates.

Antibiotic	Strain	Concentration of Ampicillin (mg/L) in Strips														
		0.016	0.032	0.064	0.125	0.25	0.5	1	2	4	8	16	32	64	128	256
Ampicillin	*H. influenzae* (R)	-	-	-	-	-	-	-	-	-	1	1	-	-	-	-
	*H. influenzae* (S)	-	1	1	4	7	15	-	-	-	-	-	-	-	-	-

Legend: “-”—not detected.

## Data Availability

The data presented in this study are available upon reasonable request from the corresponding author.
